# Serum Endocan Levels Associated with Hypertension and Loss of Renal Function in Pediatric Patients after Two Years from Renal Transplant

**DOI:** 10.1155/2016/2180765

**Published:** 2016-12-19

**Authors:** Livia Victorino de Souza, Vanessa Oliveira, Aline Oliveira Laurindo, DelmaRegına Gomes Huarachı, Paulo Cesar Koch Nogueira, Luciana de Santis Feltran, José Osmar Medina-Pestana, Maria do Carmo Franco

**Affiliations:** ^1^Nephrology Division, School of Medicine, Federal University of São Paulo, São Paulo, SP, Brazil; ^2^Pediatrics Department, School of Medicine, Federal University of São Paulo, São Paulo, SP, Brazil

## Abstract

Endocan is an important biomarker of inflammation and endothelial dysfunction that increases in association with several chronic diseases. Few published data have described the role of endocan in pediatric renal transplant (RT) patients. We evaluated the endocan concentrations in 62 children who underwent renal transplantation and assessed their relationships with the patients' blood pressure and loss of renal function. The endocan levels were significantly elevated in the pediatric RT patients who had hypertension and a loss of renal function. We determined positive correlations between the endocan concentrations and the hemodynamic variables (systolic blood pressure: *r* = 0.416; *P* = 0.001; pulse pressure: *r* = 0.412; *P* = 0.003). The endocan levels were inversely correlated with the estimated glomerular filtration rate (*r* = −0.388; *P* = 0.003). An endocan cutoff concentration of 7.0 ng/mL identified pediatric RT patients who had hypertension and a loss of renal function with 100% sensitivity and 75% specificity. In conclusion, the endocan concentrations were significantly elevated in pediatric RT patients who had both hypertension and a loss of renal function. The correlations between the endocan levels and the hemodynamic variables and the markers of renal function strengthen the hypothesis that it is an important marker of cardiorenal risk.

## 1. Introduction

Renal transplantation is one of the most effective options for the treatment of chronic renal failure in children [1, 2]. Children who receive renal transplants (RTs) have better survival rates than children who undergo dialysis [3]. In addition, these children show improvements in the quality of their lives and their life expectancies [3, 4].

Despite a positive prognosis, concerns continue to exist about the progressive loss of renal function and the development of cardiometabolic diseases among pediatric RT recipients [5–7]. The findings from several studies have demonstrated that hypertension (HT) is a major cardiovascular comorbidity that can follow renal transplantation in pediatric patients [8–11], and the prevalence of HT among these patients ranges from 60% to 90% [8]. Some investigators have verified that the development or the persistence of HT during the posttransplant period is an important risk factor that is associated with graft loss and survival [8–11]. Indeed, a negative association between HT and the glomerular filtration rate (GFR) was determined after renal transplantation in children [9]. While several factors may explain this association, the underlying mechanism remains unclear. It is possible that posttransplant HT together with donor and recipient factors, including the time on dialysis, immunosuppressive therapy, the timing of the transplantation, and the donor's age, converge to negatively impact upon the GFR.

The findings from recent research indicate that endocan could be an important predictive marker of arterial HT and renal failure [12–14]. Endocan is a soluble dermatan sulfate proteoglycan that is expressed by the human endothelial cells that are present in many different vascular beds [15, 16]. Its expression is regulated by inflammatory cytokines that induce the upregulation of endocan messenger ribonucleic acid, and the molecule is subsequently released by the endothelial cells [17]. Several reports indicate that the endocan concentrations negatively impact upon the severity of illnesses and the clinical outcomes [14, 18, 19]. The purpose of this study was to analyze the endocan concentrations in pediatric patients during the 6–24-month period after renal transplantation. We also assessed the relationships between the endocan levels and the patients' blood pressure and loss of renal function.

## 2. Methods

This study was conducted at the Renal Transplant Unit of the Nephrology Division from Kidney & Hypertension Hospital (Federal University of São Paulo; UNIFESP-EPM, São Paulo, Brazil) on pediatrics patients, who were recruited between August 2013 and July 2014. The study was carried out on 62 RT children (43 boys and 19 girls). Inclusion criteria were as follows: RT patients of either sex; patients who were between 6 and 24 months after transplant. On the other hand, patients were excluded for the following reasons: presence of systemic infection or acute rejection clinically diagnosed and biopsy proven. None of the children who underwent renal transplant received vitamin D supplementation. All patients provided a blood sample, which was collected in the morning, following an overnight fast. After that, the body weight and height were measured using a standard balance beam scale. The local ethics committee approved the study protocol* (Protocol Number: 354.875)*. All parents and children signed written informed consent/assent forms.

### 2.1. Measurement of Blood Pressure Levels

Systolic (SBP) and diastolic (DBP) blood pressure were measured with appropriate cuff size by auscultation after the child was seated for 10 min. We defined HT in accordance with the Fourth National Task Force on High Blood Pressure in Children and Adolescents [20]. An HT diagnosis was established when three or more assessments of the SBP and/or the DBP on different days over a 21-day interval were above the 95th percentiles at 6 months after transplant. We calculated the pulse pressure (PP) using the following formula: PP = SBP − DBP.

### 2.2. Renal Function Assay

The serum creatinine (sCr) levels were measured using an automated picric acid assay and a Hitachi 717 analyzer in accordance with the manufacturers' instructions. The estimated GFR (eGFR) was determined based on the sCr levels using the Bedside Schwartz equation, as follows: (eGFR = 0.413 × height (cm)/Scr [mg/dL] = mL/min/1.73 m^2^) [21].

### 2.3. Endocan Measurement

The serum endocan levels were measured using a magnetic bead-based immunoassay kit (HCVD1MAG-67K–1 Plex; Merck Millipore, Billerica, MA, USA), according to the manufacturer's protocol. The assay's concentration range was 0.02–9.6 ng/mL. The intra-assay and interassay coefficients of variation for the endocan assay were <2.43% and <5.57%, respectively. Neither significant cross-reactivity nor interference between human endocan and the breakdown product, which is a p14 peptide fragment, has been reported.

### 2.4. Statistical Analysis

The categorical variables are presented as the frequencies and the percentage distributions. The continuous variables were assessed for normality before the data were analyzed, and they are summarized as the means, standard deviations, and the 95% confidence intervals (CIs). The analyses were performed by stratifying the pediatric RT patients according to the chronic kidney disease (CKD) cutoff point, namely, an eGFR of <60 mL/min/1.73 m^2^, and the presence or absence of HT [20, 22]. To examine the effects of HT and CKD on the endocan levels, we performed a two-way analysis of variance (ANOVA) followed by pairwise multiple comparisons using the Bonferroni test that examined the significance of the main effect and/or the interactions between HT and CKD. Correlations between the continuous variables were determined using Pearson's correlation coefficient. Furthermore, logistic regression analyses were performed. Variables that showed a tendency towards a correlation and had a value of *P* < 0.20 in the univariate model were included in the multivariate analysis. A receiver operating characteristic (ROC) curve was applied to identify the best endocan cutoff point. All of the statistical tests were two-tailed, and the significance level was set at *P* < 0.05. The statistical analyses were performed using IBM®SPSS® software (Version 22, IBM Corporation, Armonk, NY, USA).

## 3. Results

Our study cohort comprised 62 pediatric RT patients, and 67% of the patients were boys. The mean age of the recipients at transplantation was 12.8 years (range: 3–17 years), and the mean age of the donors was 12.7 years (range: 2–43 years). Six of the donors were adults and 56 donors were younger than 18 years of age. The causes of renal failure were defined as uropathy in 22.6% and glomerulonephritis in 17.7% of the patients, and the etiologies were undetermined or the renal failure was associated with other causes in 59.7% of the RT patients (Table 1). After renal transplantation, 29 of 62 (46.8%) children had CKD, which was defined as an eGFR < 60 mL/min/1.73 m^2^. HT was detected in 44 of the children before renal transplantation, and HT persisted in 29 of the children after transplantation. Sixteen children had both HT and CKD after renal transplantation, and 20 children had neither HT nor CKD. Seventeen children (26%) were given steroid-free immunosuppressive therapy and seven children (25%) were given single agent antihypertensive therapy that comprised calcium channel blockers (CCBs). Demographic, anthropometric, and clinical data are presented in Tables 1 and 2.

**Figure 1:**
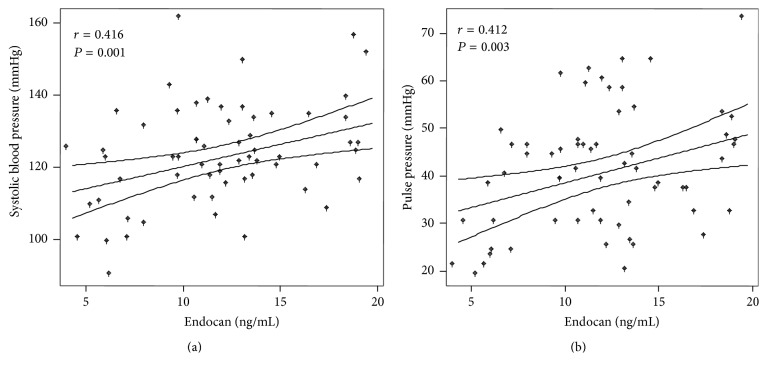
Scatter plots showing the correlations between endocan with (a) systolic blood pressure (*r* = 0.416; *P* = 0.001) and endocan with (b) pulse pressure (*r* = 0.412; *P* = 0.003). The lines represent the weighted regression with its 95% confidence interval. Statistical analysis: Pearson's correlation method.

The mean endocan concentration was 12.2 ng/mL (range: 4.3–15.4 ng/mL), and there was no significant difference between the genders with respect to the endocan concentration (*P* = 0.252). There were no correlations between the endocan levels and age (*r* = 0.120; *P* > 0.05) or the body mass index (BMI) (*r* = 0.107; *P* > 0.05). We found positive correlations between the endocan levels and the SBP (*r* = 0.416; *P* = 0.001) and the PP (*r* = 0.412; *P* = 0.003) (Figures 1(a) and 1(b)). These correlations remained significant after adjusting for gender, age, BMI, and the time on chronic dialysis (SBP: *r* = 0.333; *P* = 0.018 and PP: *r* = 0.358 and *P* = 0.011). The serum endocan levels did not change significantly when immunosuppressive or antihypertensive agents were administered (both *P* > 0.05). Inverse correlations were detected between the eGFR and the SBP (*r* = −0.274; *P* = 0.037) and the PP (*r* = −0.281; *P* = 0.032). Interestingly, endocan levels were inversely correlated with the eGFR (*r* = −0.388; *P* = 0.003).

**Figure 2:**
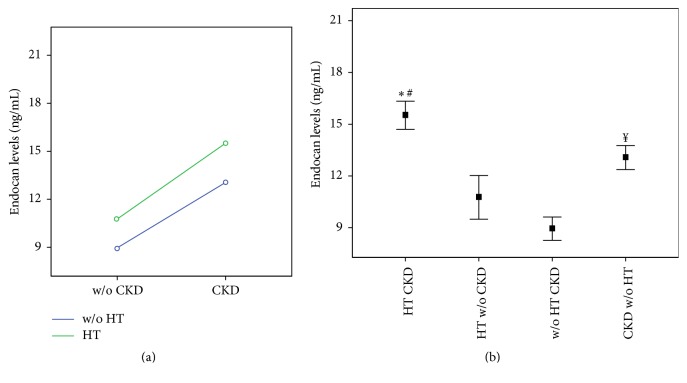
(a) Interaction plot of the mean endocan levels in RT children (*n* = 62) according to presence or not of the hypertension (HT) and/or chronic kidney disease (CKD). Statistical analysis: two-way analysis of variance (ANOVA) method. (b) Changes in the endocan levels in RT children with both HT and CKD (HTCKD; *n* = 16), with HT without CKD (HT w/o CKD; *n* = 13), with CKD without HT (CKD w/o HT, *n* = 13), and without both conditions (w/o HTCKD; *n* = 20). The bars indicate standard error of mean. Statistical analysis: two-way analysis of variance (ANOVA) followed by pairwise multiple comparison (Bonferroni test) method. ^*∗*^*P* < 0.001 versus w/o HTCKD; ^#^*P* = 0.002 versus HT w/o CKD; ^*¥*^*P* = 0.007 versus w/o HTCKD.

The two-way ANOVA revealed the significant effects of HT (*F* = 5.989; *P* = 0.017) and CKD (*F* = 25.959; *P* < 0.001) on the serum endocan concentrations (Figure 2(a)). Pediatric RT patients with HT and CKD (*n* = 16) had a significantly higher mean serum endocan concentration (15.4 ng/mL; 95% CI: 13.8–17.2) compared with that in the pediatric RT patients who did not have either of these conditions (*n* = 20) (8.9 ng/mL; 95% CI: 7.4–10.5) (*P* < 0.001) and the mean serum endocan concentration in those with HT only (*n* = 13) (10.8 ng/mL; 95% CI: 8.9–12.6) (*P* = 0.002). There was no significant difference with respect to the mean serum endocan concentration between the pediatric RT patients with HT and CKD and those with CKD only (*n* = 13) (13.1 ng/mL; 95% CI: 11.2–14.9) (*P* = 0.342) (Figure 2(b)). The mean serum endocan concentration in the pediatric RT patients with only CKD was significantly higher compared with that in the pediatric RT patients who did not have HT or CKD (*P* = 0.007) (Figure 2(b)).

**Figure 3:**
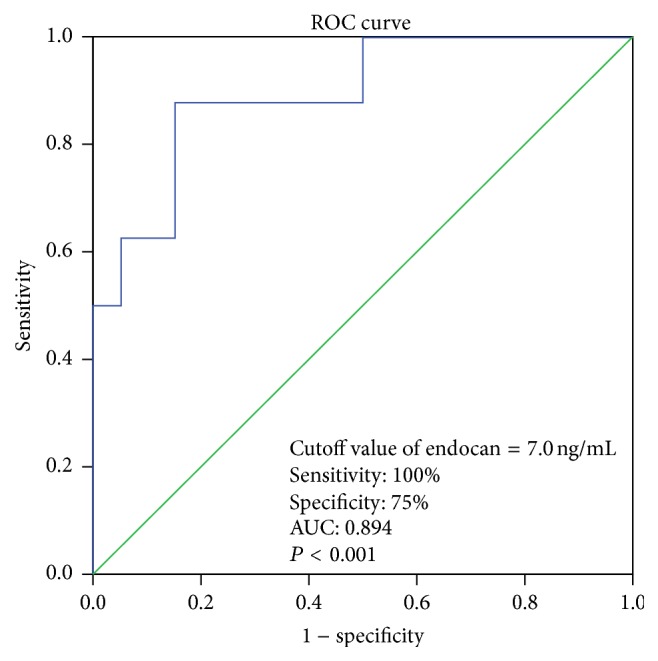
Receiver operating characteristic (ROC) curve of endocan levels predicting presence of both hypertension and loss renal function in RT children.

We performed logistic regression analyses to identify the risk factors associated with HT and the loss of renal function in the pediatric RT patients. The univariate analysis showed that donor age, a male donor, the PP, and the use of prednisone tended to be associated with these dependent variables (*P* < 0.20) (Table 3). The endocan levels were independently associated with the presence of HT and the loss of renal function in the pediatric RT patients (Table 3). The multivariate logistic regression analysis determined that only the endocan levels were independently associated with the presence of HT and the loss of renal function in our study population (Table 3). The ROC curve analysis demonstrated that an endocan cutoff concentration of 7.0 ng/mL could identify pediatric RT patients with both HT and the loss of renal function with a sensitivity of 100% and a specificity of 75% (area under the curve:  0.894; standard error: 0.053; 95% CI: 0.790–0.998; *P* < 0.001) (Figure 3).

## 4. Discussion

The main finding of present study is that serum endocan levels were significantly elevated in RT children with both HT and loss of renal function and that the serum endocan concentration was an independent predictor of the presence of HT and a loss of renal function in pediatric RT patients, after adjusting for multiple confounders. In addition, we found positive correlation of endocan with SBP and pulse pressure, and the serum levels of this biomarker were inversely correlated with eGFR among RT children.

Endocan is a soluble proteoglycan that is detected in the blood and is expressed by endothelial cells of the vasculature, lung, and kidney [16, 17]. There is strong evidence to support its role in several chronic diseases and that suggests that it is an important biomarker of endothelial function [13, 18, 19]. The findings from a recent study showed that patients with type 2 diabetes who had microalbuminuria had lower endocan levels [23], and the investigators suggested that higher levels of endocan may be present in the early phase of diabetic nephropathy and that the levels of endocan decline as the disease progresses [23]. To date, few studies have evaluated the role of the endocan levels in RT patients. The findings from a study by Li et al. [24] showed that the circulating endocan level could be used as a marker of acute rejection in RT patients. In another report, high levels of endocan were correlated with different stages of the CKD in RT patients [25]. These authors also observed that GFR loss was greater in the group with higher serum endocan levels [25]. In addition, negative correlations have been described between the endocan levels and the eGFR and endothelial function in patients with CKD who had not undergone hemodialysis or peritoneal dialysis [13]. Our results concur with and extend the findings from these studies, and they link high endocan levels with HT and GFR reductions in RT patients. Another interesting finding was the positive association between the endocan levels and the PP. Furthermore, we found that pediatric RT patients who had HT and a loss of renal function also had higher PPs compared with the pediatric RT patients who did not have these conditions. The PP elevations observed in these children might indicate the presence of arterial stiffness that leads to adverse cardiovascular outcomes. The impairment of the elastic properties of the vasculature is widely regarded as a factor that could contribute to the development and/or the persistence of HT and CKD progression [26]. Interestingly, endocan is involved in the development of vascular tissue under physiological and pathological conditions [27–30]. An important consideration is that treatment with antihypertensive and/or immunosuppressive agents may have an effect on the endocan levels. Some drugs, including angiotensin II receptor blockers, tacrolimus, and CCBs, promote alterations in endothelial function that could also influence the circulating endocan levels [31, 32]. In the current study, there were no significant changes in the serum endocan levels that were associated with the administration of immunosuppressive or antihypertensive therapy. Therefore, therapeutic scheme in the RT patient should be taken into account which makes the interpretation of endocan data among these patients even more difficult.

Some mechanisms could explain the high endocan levels in the pediatric RT patients with HT and the loss of renal function. The findings from recent studies have shown links between the endocan levels and endothelial dysfunction and inflammation [13, 18, 19]. Endocan is expressed by the endothelium, and in response to endothelial damage or the presence of inflammatory cytokines, including tumor necrosis factor-alpha and interleukin-1 beta, the endothelial cells upregulate the expression and secretion of endocan [15–17]. Endocan may stimulate the proliferation and migration of vascular smooth muscle cells [33]. Since endothelial dysfunction and inflammatory factors are associated with the development of HT and other cardiovascular diseases in RT patients, it is possible that the presence of these conditions could, at least in part, reflect the processes that were involved in increasing the endocan levels in our study population. Moreover, the increase in the endocan levels in patients with CKD may be a consequence of a reduction in its renal clearance [13]. We did not collect urine samples from the patients in the present study; therefore, we could not compare the fractional excretions of endocan in the pediatric RT patients. However, based on data recently reported in the literature, the high endocan levels in the pediatric RT patients do not appear to have been caused by reductions in the renal clearance of endocan, which results in high concentrations of endocan in the plasma [31]. Endocan is a negatively charged 50 kDa proteoglycan; therefore, under physiological conditions it cannot pass through the glomerular filtration barrier [17, 31]. Indeed, recent study found that endocan was undetectable or was present at very low levels in urine samples from healthy individuals [31]. The same investigators also found that both the plasma and the urinary concentrations of endocan were high in patients with immunoglobulin A nephropathy, which suggests that the presence of glomerular injury that involves the disruption of the basement membrane in patients with pathological renal conditions could promote endocan excretion into the urine [31]. Thus, more knowledge about renal handling of endocan is required to help us understand the mechanisms involved in the production of endocan and its clearance from the circulation via the kidney in RT patients.

The limitations of the present study include the small number of patients recruited, its cross-sectional design, and the single rather than multiple measurements of the serum endocan concentrations. In conclusion, pediatric RT patients with both HT and a loss of renal function have elevated endocan levels. The presence of correlations between the endocan levels and the SBP, the PP, and the eGFR strengthens the hypothesis that endocan is an important marker of cardiorenal risk. Although the pathological implications are not completely understood, the data from this study may help to explain the kidney damage and the increased risks of HT and other cardiovascular diseases that occur in pediatric RT patients. Further studies are necessary to clarify the importance of these correlations and to elucidate the clinical significance of endocan.

## Figures and Tables

**Table 1:** Clinical characteristics of the study population at renal transplant.

Characteristic	
Age at transplantation (years)	12.8 (3.51)
	(11.9–13.8)
Male gender	43 (69)
Causes of chronic kidney disease	
Glomerulonephritis	11 (17.7)
Uropathy	14 (22.6)
CAKUT	10 (16)
Recurrent urinary tract infections	2 (3)
Hemolytic-uremic syndrome	2 (3)
Multicystic dysplastic kidney	2 (3)
Nephropathic cystinosis	1 (1.6)
Undetermined cause	20 (33.1)
Mode of dialysis	
CAPD	16 (26)
HD	35 (56)
CAPD + HD	6 (10)
No dialysis	5 (8)
Time on chronic dialysis (months)	15.8 (9.66) (12.9–18.7)
Preexisting hypertension	44 (55)
Deceased donor	60 (96.8)
Male donor	40 (66)
Donor age	12.7 (8.7) (10.3–15.1)
Acute rejection	9 (15)
Delayed graft function	22 (36)
Cold ischemia time (min)	1265.6 (360.8) (1158.4–1372.7)
Renal artery stenosis	2 (3.2)

Data are reported as number with percent in parentheses or mean with standard deviation and 95% confidence interval in parentheses. CAKUT: congenital anomalies of the kidney and the urinary tract; HD: hemodialysis; CAPD: continuous ambulatory peritoneal dialysis.

**Table 2:** Characteristics of the study population after transplantation.

Characteristic	
Age (years)	14.5 (3.3) (13.6–15.4)
Height (cm)	149.5 (16.6) (144.9–154.1)
Weight (Kg)	45.5 (16.2) (41.0–49.6)
BMI (Kg/m^2^)	19.9 (4.5) (18.6–21.2)
SBP (mmHg)	125 (13.5) (121–128)
DBP (mmHg)	83 (12.4) (80–87)
PP (mmHg)	42 (13.2) (38–45)
sCr (mg/dL)	1.01 (0.28) (0.93–1.09)
eGFR (mL/min/1.73 m^2^)	64.3 (17.6) (59.4–69.2)
Endocan (ng/mL)	12.5 (4.2) (11.3–13.6)
Immunosuppressive therapy	
TAC + MMF + AZA	5 (8)
AZA + MMF	12 (18)
PRED + AZA + CSA	4 (6)
PRED + TAC + AZA	32 (53)
PRED + TAC	9 (15)
Antihypertensive therapy	
ACE-I + CCBs	4 (14)
ACE-I + ARBs	5 (18)
ARBs + CCBs + *β*-blockers	4 (14)
Diuretics + CCBs + *β*-blockers	8 (29)
CCBs	7 (25)

Data are reported as number with percent in parentheses or mean with standard deviation and 95% confidence interval in parentheses. BMI: body mass index; SBP: systolic blood pressure; DBP: diastolic blood pressure; PP: pulse pressure; sCr: serum creatinine; eGFR: glomerular filtration rate estimated by creatinine; TAC: tacrolimus; MMF: mycophenolate mofetil; AZA: azathioprine; CSA: cyclosporine A; PRED: prednisone; ACE-I: ACE inhibitors; CCBs: calcium channel blockers; ARBs: angiotensin receptor blockers.

**Table 3:** Logistic regression analysis for the presence of hypertension and chronic kidney disease (CKD) in RT children.

Variables	Univariate regression		Multivariate regression	
	Analysis		Analysis	
	OR (95% CI)	*P* value	OR (95% CI)	*P* value
Age (per years)	1.110 (0.884–1.395)	0.369		
Male gender (no/yes)	1.333 (0.269–5.606)	0.725		
BMI (*per* Kg/m^2^)	1.066 (0.902–1.260)	0.456		
Pulse pressure (mmHg)	1.084 (1.015–1.157)	0.016	1.079 (0.969–1.202)	0.166
Endocan (per ng/mL)	1.855 (1.187–2.898)	0.007	2.070 (1.097–3.907)	0.035
Donor age (*per *year)	0.832 (0.708–0.978)	0.086	0.839 (0.659–1.085)	0.317
Male donor (no/yes)	1.367 (0.860–1.767)	0.176	1.317 (0.448–4.577)	0.399
Preexisting hypertension (no/yes)	4.437 (0.449–9.723)	0.224		
Chronic dialysis (*per* months)	1.021 (0.932–1.120)	0.652		
Delayed graft function (no/yes)	1.592 (0.382–6.625)	0.523		
Cold ischemia time (*per *min)	1.048 (0.258–4.256)	0.948		
Prednisone (no/yes)	1.640 (0.168–2.436)	0.198	1.486 (0.743–5.987)	0.567
Tacrolimus (no/yes)	2.213 (0.467–7.238)	0.298		
Mycophenolate mofetil (no/yes)	1.201 (0.221–5.521)	0.833		
Azathioprine (no/yes)	0.400 (0.109–1.254)	0.251		

Data are reported as odds ratio (OR) and 95% confidence interval (95% CI).

## References

[1] Warady B. A., Hébert D., Sullivan E. K., Alexander S. R. (1997). Renal transplantation, chronic dialysis and chronic renal insufficiency in children and adolescents. The 1995 annual report of the North American Pediatric Renal Transplant Cooperative study. *Pediatric Nephrology*.

[2] van der Heijden B. J., van Dijk P. C. W., Verrier-Jones K., Jager K. J., Briggs J. D. (2004). Renal replacement therapy in children: data from 12 registries in Europe. *Pediatric Nephrology*.

[3] McDonald S. P., Craig J. C. (2004). Long-term survival of children with end-stage renal disease. *The New England Journal of Medicine*.

[4] Mehrabi A., Kashfi A., Tönshoff B. (2004). Long-term results of paediatric kidney transplantation at the University of Heidelberg: a 35 year single-centre experience. *Nephrology Dialysis Transplantation*.

[5] Wigger M., Drückler E., Muscheites J., Stolpe H. J. (2001). Course of glomerular filtration rate after renal transplantation and the influence of hypertension. *Clinical Nephrology*.

[6] Büscher R., Vester U., Wingen A.-M., Hoyer P. F. (2004). Pathomechanisms and the diagnosis of arterial hypertension in pediatric renal allograft recipients. *Pediatric Nephrology*.

[7] Seeman T. (2009). Hypertension after renal transplantation. *Pediatric Nephrology*.

[8] Opelz G., Wujciak T., Ritz E. (1998). Association of chronic kidney graft failure with recipient blood pressure. *Kidney International*.

[9] Moudgil A., Martz K., Stablein D. M., Puliyanda D. P. (2010). Variables affecting estimated glomerular filtration rate after renal transplantation in children: a NAPRTCS data analysis. *Pediatric Transplantation*.

[10] Mitsnefes M. M., Khoury P. R., McEnery P. T. (2003). Early posttransplantation hypertension and poor long-term renal allograft survival in pediatric patients. *Journal of Pediatrics*.

[11] Groothoff J. W., Cransberg K., Offringa M. (2004). Long-term follow-up of renal transplantation in children: a Dutch cohort study. *Transplantation*.

[12] Afsar B., Takir M., Kostek O., Covic A., Kanbay M. (2014). Endocan: a new molecule playing a role in the development of hypertension and chronic kidney disease?. *Journal of Clinical Hypertension*.

[13] Yilmaz M. I., Siriopol D., Saglam M. (2014). Plasma endocan levels associate with inflammation, vascular abnormalities, cardiovascular events, and survival in chronic kidney disease. *Kidney International*.

[14] Lee H. G., Choi H. Y., Bae J. (2014). Endocan as a potential diagnostic or prognostic biomarker for chronic kidney disease. *Kidney International*.

[15] Béchard D., Gentina T., Delehedde M. (2001). Endocan is a novel chondroitin sulfate/dermatan sulfate proteoglycan that promotes hepatocyte growth factor/scatter factor mitogenic activity. *The Journal of Biological Chemistry*.

[16] Zhang S. M., Zuo L., Zhou Q. (2012). Expression and distribution of endocan in human tissues. *Biotechnic and Histochemistry*.

[17] Lassalle P., Molet S., Janin A. (1996). ESM-1 is a novel human endothelial cell-specific molecule expressed in lung and regulated by cytokines. *The Journal of Biological Chemistry*.

[18] Balta S., Mikhailidis D. P., Demirkol S., Ozturk C., Celik T., Iyisoy A. (2015). Endocan: a novel inflammatory indicator in cardiovascular disease?. *Atherosclerosis*.

[19] Balta S., Mikhailidis D. P., Demirkol S. (2014). Endocan-a novel inflammatory indicator in newly diagnosed patients with hypertension: a pilot study. *Angiology*.

[20] National High Blood Pressure Education Program Working Group on High Blood Pressure in Children and Adolescents (2004). The fourth report on the diagnosis, evaluation, and treatment of high blood pressure in children and adolescents. *Pediatrics*.

[21] Schwartz G. J., Work D. F. (2009). Measurement and estimation of GFR in children and adolescents. *Clinical Journal of the American Society of Nephrology*.

[22] Levey A. S., Coresh J., Balk E. (2003). National Kidney Foundation practice guidelines for chronic kidney disease: evaluation, classification, and stratification. *Annals of Internal Medicine*.

[23] Cikrikcioglu M. A., Erturk Z., Kilic E. (2016). Endocan and albuminuria in type 2 diabetes mellitus. *Renal Failure*.

[24] Li S., Wang L., Wang C. (2012). Detection on dynamic changes of endothelial cell specific molecule-1 in acute rejection after renal transplantation. *Urology*.

[25] Su Y.-H., Shu K.-H., Hu C.-P. (2014). Serum endocan correlated with stage of chronic kidney disease and deterioration in renal transplant recipients. *Transplantation Proceedings*.

[26] Peralta C. A., Jacobs D. R., Katz R. (2012). Association of pulse pressure, arterial elasticity, and endothelial function with kidney function decline among adults with estimated GFR >60 mL/min/1.73 m^2^: the multi-ethnic study of atherosclerosis (MESA). *American Journal of Kidney Diseases*.

[27] Carrillo L. M., Arciniegas E., Rojas H., Ramírez R. (2011). Immunolocalization of endocan during the endothelial-mesenchymal transition process. *European Journal of Histochemistry*.

[28] Delehedde M., Devenyns L., Maurage C.-A., Vivès R. R. (2013). Endocan in cancers: a lesson from a circulating dermatan sulfate proteoglycan. *International Journal of Cell Biology*.

[29] Icli A., Cure E., Cure M. C. (2016). Endocan levels and subclinical atherosclerosis in patients with systemic lupus erythematosus. *Angiology*.

[30] Altintas N., Mutlu L. C., Akkoyun D. C. (2016). Effect of CPAP on new endothelial dysfunction marker, endocan, in people with obstructive sleep apnea. *Angiology*.

[31] Lee Y. H., Kim J. S., Kim S. (2016). Plasma endocan level and prognosis of immunoglobulin A nephropathy. *Kidney Research and Clinical Practice*.

[32] Celik T., Balta S., Karaman M. (2015). Endocan, a novel marker of endothelial dysfunction in patients with essential hypertension: comparative effects of amlodipine and valsartan. *Blood Pressure*.

[33] Menon P., Kocher O. N., Aird W. C. (2011). Endothelial cell specific molecule-1 (ESM-1), a novel secreted proteoglycan stimulates vascular smooth muscle cell proliferation and migration. *Circulation*.

